# Deep Modular Bilinear Attention Network for Visual Question Answering

**DOI:** 10.3390/s22031045

**Published:** 2022-01-28

**Authors:** Feng Yan, Wushouer Silamu, Yanbing Li

**Affiliations:** 1 School of Information Science and Engineering, Xinjiang University, Urumqi 830046, China; yanfeng@stu.xju.edu.cn (F.Y.); liyb@xju.edu.cn (Y.L.); 2 Laboratory of Multi-Lingual Information Technology, Xinjiang University, Urumqi 830046, China

**Keywords:** attention mechanism, visual question answering, multi-model, bilinear attention network

## Abstract

VQA (Visual Question Answering) is a multi-model task. Given a picture and a question related to the image, it will determine the correct answer. The attention mechanism has become a de facto component of almost all VQA models. Most recent VQA approaches use dot-product to calculate the intra-modality and inter-modality attention between visual and language features. In this paper, the BAN (Bilinear Attention Network) method was used to calculate attention. We propose a deep multimodality bilinear attention network (DMBA-NET) framework with two basic attention units (BAN-GA and BAN-SA) to construct inter-modality and intra-modality relations. The two basic attention units are the core of the whole network framework and can be cascaded in depth. In addition, we encode the question based on the dynamic word vector of BERT(Bidirectional Encoder Representations from Transformers), then use self-attention to process the question features further. Then we sum them with the features obtained by BAN-GA and BAN-SA before the final classification. Without using the Visual Genome datasets for augmentation, the accuracy of our model reaches 70.85% on the test-std dataset of VQA 2.0.

## 1. Introduction

**Figure 1:**
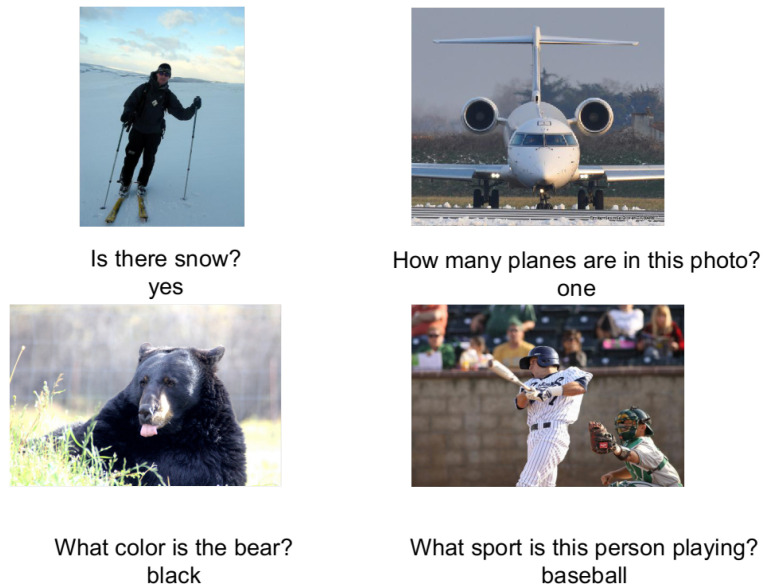
Examples of training questions and their correct answer from the VQA v2 dataset.

The task goal of VQA (Visual Question Answering) [1] is to build a question answering system like human intelligence, which can recognize the category, spatial relationship, and other information of objects from the specified pictures. VQA has broad application scenarios and has far-reaching significance for the development of artificial intelligence (see Figure 1).

Our model can be applied to the blind assistant robot. The surrounding images and audio can be obtained through the robot’s hardware sensor as the input of our model, which can effectively help the blind perceive the surrounding objects.

The most challenging problem in VQA is establishing the association between each region in the image and the words in the question, and the model of VQA has the ability to align the image and text semantically. VQA models not only have to understand the content of a picture, but also have to find the corresponding answer to the question, which presents a greater challenge to the model and makes it more intelligent.

MCB [2], MFB [3], and Mutan [4] capture the high-level interactions between question and images features based on the fusion method. However, the scope of use is limited, and it is not easy to apply to other VQA models.

Attention mechanisms [5,6,7,8] are very important for deep learning, and it has successfully been applied to the VQA task. The model based on the attention mechanism focuses on the key information. Aderson et al. [9] proposed a bottom-up and top-down attention mechanism and won the VQA Challenge 2017. They use the concatenated attention mechanism to get the image attention guided by the question. However, the model ignores the relationship between each word and image region. BAN [10] focuses on exploring the inter-modality relations between word–region pairs, ignoring the intra-modality relations.

MCAN [11], DFAF [12], and CMCN [13] simultaneously explore inter-modality relations and intra-modality relations, and achieve good results. MCAN proposes a deep Modular Co-Attention Network that consists of Modular Co-Attention (MCA) layers cascaded in depth.

Inspired by the MCAN, we designed two basic attention units (BAN-GA and BAN-SA), combined and cascaded. Our work attempts to use bilinear attention to construct inter-modality and intra-modality relations between visual and language features. Along with visual attention, learning textual attention is also very important. We try to use the pre-trained language model BERT [14] to encode the question. In addition, we use a self-attention unit to process the question features further. On this foundation, our model achieves better performance.

Almost all VQA models use the attention mechanism. However, compared with BUTD, we use the co-attention mechanism instead of the image-guided attention mechanism; in terms of question embedding, we use sentence vectors instead of word vectors to better express the characteristics of the question. Compared with the fusion method, we use BAN to construct the relationship between modes, and we also pay attention to the internal relationship of modes. Compared with MCAN, our model uses a bilinear attention network instead of the more customary one based on dot-products.

Finally, this paper’s contribution and innovation are summarized as follows:

- In this paper, we propose a deep multimodality attention network (DMBA-NET) framework with two basic attention units (BAN-GA and BAN-SA) to construct inter-modality and intra-modality relations between visual and language features. BAN-GA and BAN-SA are the core of the whole network framework, and they can be cascaded in depth. Unlike other models, we use bilinear attention to calculate the inter-modality and intra-modality attention instead of dot-product. Our experiments show that we obtain more refined and rich features.
- We encode text information based on the dynamic word vector of BERT. Then we use multi-head self-attention to process the text features and sum them with the features obtained in the previous step, before the final classification, which further improves the model’s accuracy, indicating that this method can work together.
- We visualize the attention of the model and the experimental results, which can help us better understand the interaction between multimodal features. Extensive ablation experiments are carried out, and the experimental results show that each module in the model can play its effectiveness.

## 2. Related Work

### 2.1. Attention

Attention mechanism [5,6,7,8] focuses on the main areas of images and questions, ignoring some irrelevant information. Various attention mechanisms have brought significant progress to VQA and become the standard configuration of the model. The attention mechanism also inspires our model. The early attention method uses the question to find the area related to the question in the images.

### 2.2. High-Level Attributes and Knowledge

Refs. [15,16,17,18] deals with visual question, answering with the help of information from external knowledge base. Answering questions requires understanding the visual content of the image, such as answering “how many mammals are there in the picture?”. First, you need to know whether the animals in the picture belong to mammals. This kind of question can only be answered with the help of external knowledge. Some studies combine VQA tasks with the knowledge base, and some datasets are specifically aimed at this kind of method, such as the kb-vqa data set and the fvqa dataset. If you want to answer complex questions, it is necessary to acquire knowledge from outside.

### 2.3. VQA Pre-Training

Most VQA methods use two separate pre-training models: visual model training on ImageNet [19] and VG [20], and word embedding for language features. Since these features of individual training may not be optimal for joint visual and language understanding, a hot topic recently is the development of joint pre-training models [21,22,23,24] for visual and language tasks.

### 2.4. Feature Fusion

In the early stage, multimodal fusion [3,4,25] was concatenation or element-wise multiplication, and Bilinear Fusion used bilinear pooling to fuse multimodal features to get the high-level interactions. However, these methods need high computational. Many approximated fusion methods, including MCB [2], MLB [26] and MUTAN [4], were proposed, which have shown better performance with fewer parameters.

## 3. Deep Modular Bilinear Attention Network

**Figure 2:**
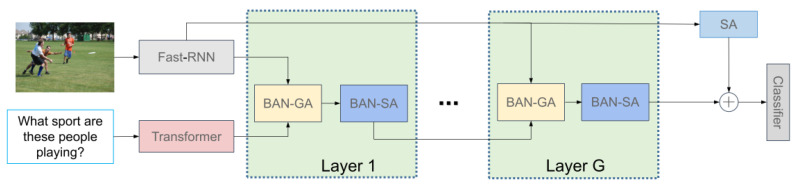
Overview of the proposed model. A deep neural network implements a joint embedding of the question feature encoded by BERT and an image feature encoded by Faster-RCNN [27]. BAN-GA denotes Multi-glimpse Bilinear Guided-Attention Network; BAN-SA denotes Multi-glimpse Bilinear Self-Attention Network; and SA represents Multi-head Self-Attention.

In this section, we elaborate on the proposed model for the VQA task. The overview of the proposed model is illustrated in Figure 2. Each layer of our model is composed of two basic units, BAN-GA and BAN-SA. BAN-SA represents the bilinear self-attention network, and BAN-GA represents the bilinear guided attention network. We will describe the composition of these two basic units in detail below.

### 3.1. Question and Image Encoding

The question of the VQA is a sequence of words. We encode it by BERT to q. The question is reduced to a maximum of 14 words. The extra words are discarded (we only deal with the first 14 words), and questions with less than 14 are filled with zero vectors.

BERT [14] is a new language representation model, the advantage of BERT is the use of the bidirectional transformer. Using the prediction target word and the next sentence, multi-task learning method was used for training. Other language expression models include word2vec [28], Glove [29], Elmo [30], GPT-2 [31]. Word2vec is a static method. Although it has strong universality, it cannot be dynamically optimized for specific tasks. Glove uses co-occurrence matrix and considers local information and overall information at the same time.

We embed these words into the 768-dimensional feature vectors using a pre-trained BERT model.

We refer to the correspondence between the word and index in the vocabulary of BERT and convert the word to index.

(1) $$I=[I_{1},I_{2},\cdots ,I_{t}]$$

where $I_{t}$ represents the index of the vocabulary of BERT at position t in the question.

(2) $$Q=BERT(I_{t})$$

where $Q\in R^{d_{q}*N}$ is the sequence of question representations. $d_{q}=768$ is the output dimension of the BERT. During training, the BERT parameters are fine-tuned using the question-answering loss.

Because of Faster R-CNN’s [27] excellent performance in various target recognition tasks, it is selected for image feature extraction in this section.

Following the conventional way, we utilize Faster-RCNN with the ResNet model to detect M objects from an image. We denote the object-level image features as $V\in R^{d_{o}*M}$. We fine-tune the Faster R-CNN detector’s last layer during training and normalize it. The calculation formula is as follows:

(3) V=RCNN(I)

where RCNN(.) represents extraction of image features through a Faster R-CNN model, $W_{1}\in R^{d_{o}*d_{o}}$ is the projection parameters and $d_{o}=2048$ is the dimension of each object feature.

### 3.2. Multi-Glimpse Bilinear Guided-Attention Network

**Figure 3:**
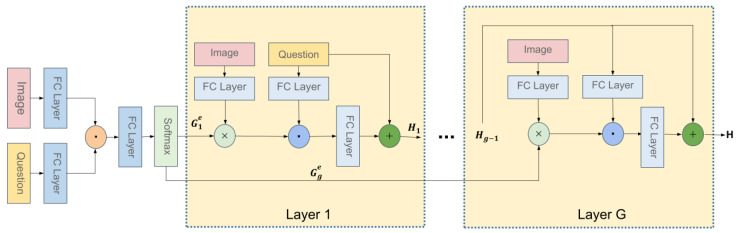
Architecture of our Multi-glimpse Bilinear Guided-Attention unit.

Here, we introduce a bilinear attention network to get the relationship between each word of the question and the region features of the image. On the one hand, the bilinear model reduces the dimension of input and reduces the amount of calculation. On the other hand, more detailed co-attention can be obtained. Figure 3 presents the multi-glimpse extensions.

We use the bilinear method to get bilinear attention map $G^{GA}$ between image and question. The calculation formula is as follows:

(4) $$G^{GA}=\mathrm{softmax}\left(\left(𝟙\cdot P_{\mathrm{GA}}^{T}\right)\;\sigma \left(Q^{T}U_{\mathrm{GA}}^{\prime }\right)\circ \sigma \left(V_{\mathrm{GA}}^{\prime T}V\right)\right)$$

where $U_{\mathrm{GA}}^{\prime }\in R^{d_{q}*K}$, $V_{\mathrm{GA}}^{\prime }\in R^{d_{o}*K}$, $P_{\mathrm{GA}}\in R^{K}$ $q_{i}\in R^{d_{q}}$, $v_{j}\in R^{d_{o}}$ are variables to be learned.

Then we use bilinear attention map $G^{GA}$ to integrate the image region feature V and the question embedding Q; the k-th joint embedding is as follows:

(5) $$\begin{array}{c} z_{k}=\sum_{i=1}^{d_{q}}\sum_{j=1}^{d_{o}}G_{i,j}^{GA}\sigma \left(q_{i}^{T}U_{\mathrm{GA}}\right)\sigma \left(V_{\mathrm{GA}}^{T}v_{j}\right) \end{array}$$

where $G_{i,j}^{GA}$ is the bilinear attention map, $U_{\mathrm{GA}}\in R^{d_{q}\times K}$, $V_{\mathrm{GA}}\in R^{d_{o}\times K}$ are variables to be learned.

For the convenience, the bilinear attention networks can be defined as follows:

(6) $$z=BAN\left(Q,V;G^{GA}\right)$$

We get multiple bilinear attention maps, and use residual to integrate them; compared with sum and concat, residual can get a better effect.

(7) $$H_{i+1}=W_{i}^{GA}BAN_{i}\left(H_{i},V;G_{i}^{GA}\right)+H_{i}$$

where $W_{i}^{GA}\in R^{d_{q}\times K}$, $H_{0}=V$, *V* and $H_{i}$ have the same size, max(i)=g, *g* is the number of glimpses. We use H to represent the output of the last glimpse, denoted as:

(8) $$H=H_{g}$$

### 3.3. Multi-Glimpse Bilinear Self-Attention Network

**Figure 4:**
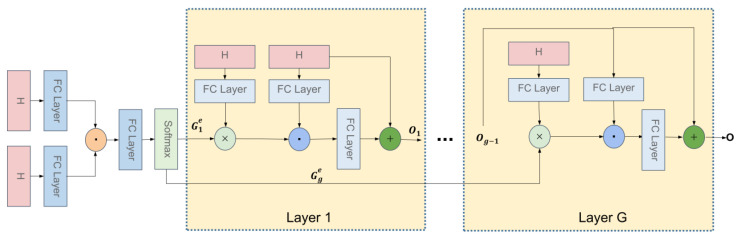
Architecture of our Multi-glimpse Bilinear Self-Attention unit.

The structure of the Bilinear Self-Attention Network is similar to the Bilinear Guided-Attention Network. The overview of the Multi-glimpse Bilinear Self-Attention Network is illustrated in Figure 4. Both input of the Bilinear Self-Attention Network are H.

After obtaining the integrated features H of the question and image, inspired by the self-attention mechanism, we further process the integrated features and get fine features. The calculation method is still using the method of BAN described above. Previously, we input two features: V and Q, and now we input a fusion feature: H. The calculation method is as follows:

(9) $$G^{SA}=\mathrm{softmax}\left(\left(𝟙\cdot P_{\mathrm{SA}}^{T}\right)\;\sigma \left(H^{T}U_{\mathrm{SA}}^{\prime }\right)\circ \sigma \left(V_{\mathrm{SA}}^{\prime T}H\right)\right)$$

where $U_{\mathrm{SA}}^{\prime }\in R^{d_{q}*K}$, $V_{\mathrm{SA}}^{\prime }\in R^{d_{q}*K}$, $P_{\mathrm{GA}}\in R^{K}$, $H\in R^{d_{K*N}}$ are variables to be learned.

The i-th output is defined as:

(10) $$O_{i}=W_{i}^{SA}BAN_{i}\left(O_{i-1},H;G_{i}^{SA}\right)+O_{i-1}$$

where $W_{i}^{SA}\in R^{d_{q}\times K}$ projects the joint embeddings to the same dimension of Q.

### 3.4. Multi-Head Self-Attention

**Figure 5:**
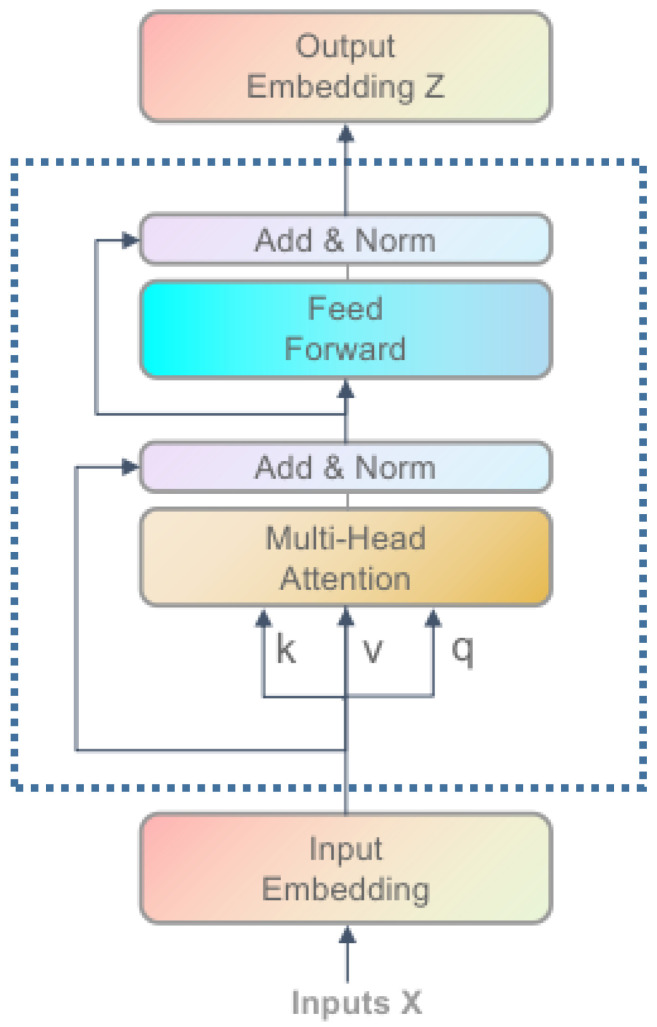
Architecture of our Multi-head Self-Attention.

After obtaining the question features from BERT, we use self-attention to process the features further. Now we introduce self-attention, illustrated in Figure 5. Self-attention has the same Q (Query), K (Key), and V (Value). First, we calculate the dot products of the query and all the keys, then divide each by d(the dimension of the question feature). Finally, we apply a softmax to get the attention weight. The calculation formula is as follows:

(11) $$F=Attn\left(Q,K,V\right)=softmax\left(\frac{QK^{T}}{\sqrt{d}}\right)V$$

where $Q,K,V\in R^{n\times d}$ is the weight matrix, *d* is the dimension of the feature, *n* is the number of the words for the question features.

To get better feature representation, we usually use a multi-head mechanism. Each head is an independent Scale Dot-Product Attention operation. The formula is as follows:

(12) $$\begin{array}{c} F=MH\_Attn\left(Q,K,V\right)=concat\left(head_{1},head_{2},...,head_{h}\right)W^{O} \end{array}$$

(13) $$head_{i}=Attn\left(W_{i}^{Q},W_{i}^{K},W_{i}^{V}\right)$$

where $W^{O}\in R^{h*d_{h}*d}$ and $W_{i}^{Q},W_{i}^{K},W_{i}^{V}\in R^{d*d_{h}}$ are learned projection matrices, *d* is the dimension of the feature, h is the number of the head, we make $d_{h}=d/h$.

We input the text feature q extracted from BERT into the multi-head attention mechanism, which can be expressed as:

(14) $$Q_{SA}=MH\_Attn\left(q,q,q\right)$$

where $Q^{SA}\in R^{d_{q}*N}$ are results after processing.

### 3.5. Feature Fusion and Answer Prediction

After getting the image feature and the question feature, we need to perform feature fusion. The image feature vector is 2048 dimensions, and the question vector is 768 dimensions. The representations of the question q and the image $\hat{v}$ are passed through linear layers and then combined with a simple Hadamard product. The calculation formula is as follows:

(15) $$S=LayerNorm(W_{a}O_{g}+W_{b}Q_{SA})$$

where $W_{a}\in R^{d_{q}*d_{q}}$ and $W_{b}\in R^{d_{q}*d_{q}}$ is the projection parameters, we use LayerNorm to stabilize training.

The resulting vector $h\in R^{d_{y}}$ is referred to as the joint embedding of the question and the image features, and is then fed to the output classifier.

After obtaining the fused feature *s*, we pass it to a two-layer MLP for classification:

(16) $$p=W_{c}\sigma \left(W_{d}S\right)$$

where $W_{c}\in R^{d_{z}\times 2C}$ and $W_{d}\in R^{2C\times C}$ is the projection parameters, $d_{z}$ is set to 3129.

### 3.6. Loss Function

We utilize the binary cross-entropy loss (BCE) as loss function to train our model, which is calculated as

(17) $$L=-[y\log\hat{y}+\left(1-y\right)\log\left(1-\hat{y}\right)]$$

where *y* is the occurrence probablility of the ground-truth answer, $\hat{y}$ is the prediction.

## 4. Experiment

### 4.1. Datasets

The VQA task has many datasets, including COCO-QA, FM-IQA, Visual Genome [20], and VQA v2 [1]. We use the VQA v2 dataset for training and testing.

The dataset is divided into train, val, and test in advance. They are composed of 248,349 questions, 121,512 questions, and 244,302 questions, respectively, of which 204k images are from the Microsoft coco dataset. All questions are divided into three types: Yes/no, count, and others. Each question has ten free answers.

There are at least three questions per picture, and on average, there are 5.4 questions per picture. Each question has ten real answers, which ten different people annotate. The people who provide the answers are not the same as the people who ask the questions. The calculation method is as follows:

(18) $$acc\left(ans\right)=min\left\{\frac{\#humans\;that\;said\;ans}{3},1\right\}$$

where ans is the answer predicted by the VQA model.

### 4.2. Experimental Setup

The dimension of image is set to $d_{o}$ = 2048. We set the dimension of the question representation $d_{q}$ to 768. The length of question t is 14. Following the approach in [10], the number of the candidate answers $d_{z}$ is set to 3129, which is determined by the minimum occurrence of the answer in a unique question as nine times. We set the glimpse number to 4, the batch size to 128, and the basic learning rate to 0.001. After the 18th epoch, reduce the learning rate to 1/10 of the previous one. Besides this, gradient client and dropout technology were used. Adamax [32], a variant of Adam, is used to optimize our model. All experiments are implemented with the Pytorch and performed on a workstation with RTX 3090 GPU.

### 4.3. Ablation Analysis

Before the ablation experiment, we compared the effects of two image feature extraction methods, one using BUTD [9] and the other using Pythia [33]. Pythia used the new state-of-the-art detectors based on feature pyramid networks (FPN) from Detectron, which uses ResNeXt as backbone and has two fully connected layers (fc6 and fc7) for region classification. We use two models to experiment on these two image features respectively. The results show that the use of Pythia image feature can improve the results, but the increase is different for different models. Using Pythia image feature, it is more suitable for BAN. Table 1 shows the results. In later experiments, we use Pythia image feature by default.

In this section, we design some ablation experiments on VQA 2.0 to verify the effectiveness of our model. For fair comparison, we feed exactly the same features to all the evaluated models that are trained on the training set and tested on the validation set.

Table 2 shows the effectiveness of the proposed components.

- BAN-GA [10]: denotes Bilinear Guided-Attention Networks.
- BAN-GA + BERT: represents Bilinear Guided-Attention Networks with BERT. We use BERT to encode the quesion features.
- BAN-GA + BAN-SA: represents Bilinear Guided-Attention Networks with Bilinear Self-Attention Networks.
- BAN-GA + Q-SA: represents Bilinear Attention Networks with Question Self-Attention Networks.
- BAN-GA + BERT + BAN-SA + Q-SA: represents our final model.

In the first line in Table 2, we only used the Bilinear Guided-Attention Networks.

In the second line, we added the BERT model based on the first line of the experiment and obtained a 1.6% improvement, which proves that the dynamic word vector can improve the model’s text representation ability.

In the third row, we used the Bilinear Guided-Attention Networks and the Bilinear Self-Attention Networks, which was improved by 1.58%, which proved the effectiveness of the Bilinear Self-Attention Networks.

In the fourth row, we added the Q-SA unit based on the first line of the experiment and obtained a 1.67% improvement, which proves that the dynamic word vector can improve the model’s text representation ability.

**Figure 6:**
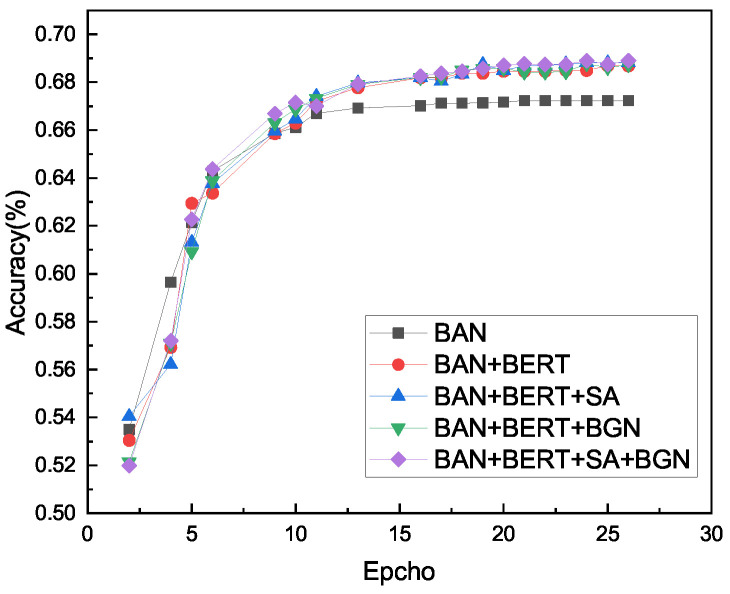
Accuracy curve of ablation model. The results are reported after using three random initialization.

**Figure 7:**
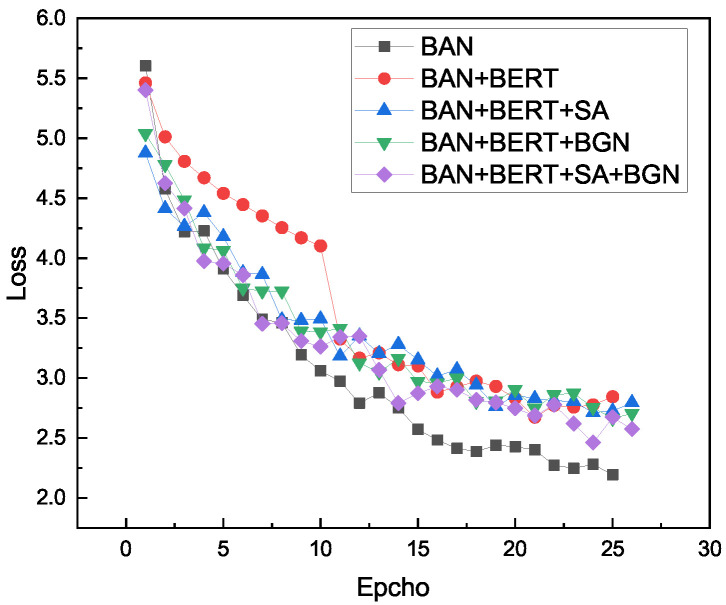
Loss curve of ablation model. The results are reported after using three random initialization.

In the last row, the accuracy of the proposed method is 69.48%. The accuracy curve and loss curve during the training process of the ablation experiment are shown in Figure 6 and Figure 7. The validity of our model is proven.

Table 3 shows the validation scores on VQA2.0 dataset for the number of glimpse of our models.

Furthermore, we studied the effect of BERT’s learning rate on our model. Table 4 shows the results of different BERT’s learning rate. When the BERT’s learning rate is set to lr × 0.001, the accuracy increases slightly, and by increasing its learning rate, it achieves the best performance at lr × 0.02. It can prove that our model is effective and compatible with BERT.

### 4.4. Qualitative Analysis

**Figure 8:**
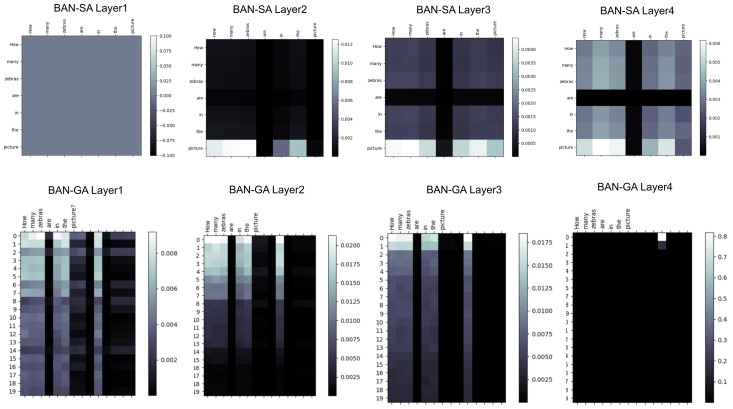
We set the number of layers of the model to 4. Visualizations of the attention maps of the BAN-SA and BAN-GA in each layer. BAN-GA and BAN-SA respectively denote Bilinear Guided-Attention Network and Bilinear Self-Attention Network.

In Figure 8, we visualize the attention maps of the BAN-SA and BAN-GA in each layer. It is found from the figure that the important part of the question cannot be found in the first layer of BAN-SA. With the increase of layers, we can intuitively see which words have a large weight in the last layer. In the attention map of BAN-SA, the words ‘how’, ‘many’, and ‘zebras’ get large attention weights. It can be explained that we have found keywords from the question.

**Figure 9:**
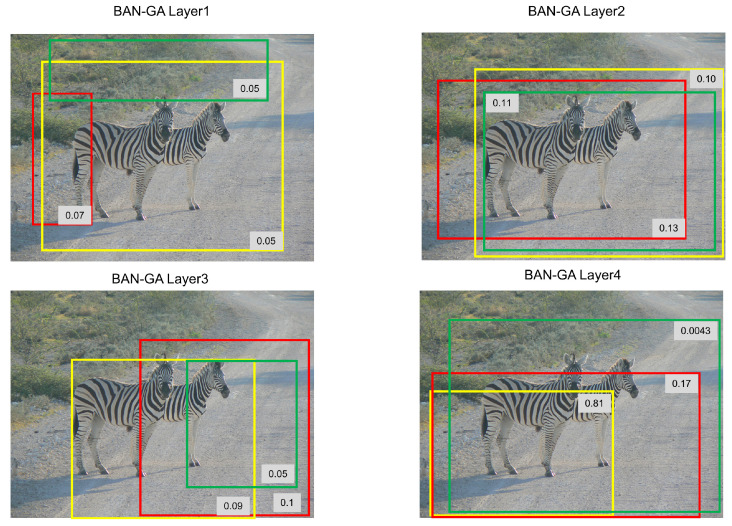
We set the number of layers of the model to 4. Visualizations of the top three image regions with the largest weight, marked the corresponding regions with boxes, and the numbers on the boxes represent the weight of the corresponding image regions in each layer. With the increase of the number of layers, the weight of the top two regions increases gradually.

For the attention maps of BAN-GA, in Figure 9, we can see from the first three layers that the corresponding information between the question and the image region is found because this question is a number of types. The last layer shows two areas with large weight, and the weight of other areas is particularly low, which exactly corresponds to the correct answer, ‘Two’. Through the visualization of other amount questions, we find that the features of the BAN-GA in the last layer will have a large weight in one column.

For the 100 regions in the image, we select the top three largest weight for visualization, mark the corresponding regions with boxes, and the numbers on the boxes represent the corresponding weights of the image regions. We can intuitively find that the boxes basically frame the two zebras in the image corresponding to the question.

### 4.5. Comparison with the State-of-the-Art

In this section, we compare our model with the state-of-the-art models on VQA 2.0 datasets. Table 5 shows the evaluation results on VQA 2.0 test-dev dataset; all models are based on a single model.

Among them, BUTD [9] proposed the Bottom-Up attention method and won the VQA Challenge 2017. Compared with this model, we improved the accuracy by 5.37%. MFB [3] and MFH [25] are based on the bilinear pooling method. Our model outperforms them. In addition, the Counter model focuses on the number of question of VQA, and our model is 2.6% higher than Counter. The MuRel [35] model is a multimodal relational network that is learned end-to-end to reason over images. Our model increases the overall accuracy of MuRel by 2.66% on the test-dev set. The MRA-Net [36] model explores both textual and visual relations to improve performance and interpretability. Our model is 1.67% higher than MRA-Net. The results demonstrate our model has a certain reasoning ability. MCAN [11] propose a deep Modular Co-Attention Network that consists of Modular Co-Attention (MCA) layers cascaded in depth. Our model achieves considerable performance without using the Visual Genome datasets. Different from DFAF and CMCN, we use bilinear attention to calculate the inter-modality and intra-modality attention instead of dot-product. From the experimental comparison, our model is more effective.

**Figure 10:**
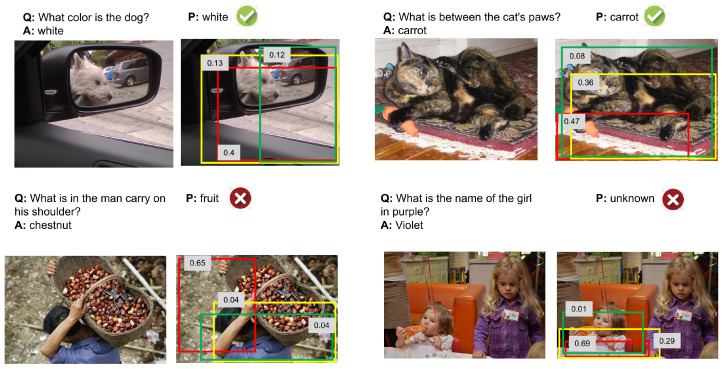
The qualitative evaluation and typical examples of the images region attentions of our model. The top row examples are the correct predictions, while the bottom row shows two incorrect predictions. For each example, we select the top 3 largest image region weight for visualization, mark the corresponding regions with boxes, and the numbers on the boxes represent the corresponding weights of the image regions.

To better prove the effects of the image attentions, we randomly picked from different question types and visualized the attentions in Figure 10. Most of the top three regions with the highest probability in the box are related to questions. The image attentions are focused on the keyword of the questions. From this point of view, our model is effective. From the incorrect examples, the first wrong prediction shows that our model is not good at recognizing some uncommon objects, indicating that the training samples are insufficient and do not cover some uncommon and rare things. The second wrong prediction shows that the model is not good at text recognition in the image (e.g., name of the girl in the fourth example), which provides a good idea for us to improve the accuracy of the model in the later stage. In the future, we can consider adding the OCR function to improve the ability of the model to recognize text. These weaknesses are helpful to guide further improvements for VQA.

## 5. Conclusions

VQA task is a very serious challenge in the field of computer vision, and it has a very wide application prospect. In this paper, we proposed a framework that can obtain more refined visual and text representation and design two basic attention units (BAN-GA and BAN-SA) to explore the inter-modality and intra-modality relations, which can be cascaded in depth. In addition, we encoded the question based on the dynamic word vector of BERT. We used multi-head self-attention to process the question features and summed them with the features obtained by the BAN-GA and BAN-SA, which further improved the model’s accuracy.

From the incorrect examples in Figure 10, in the future, we intend to focus on the research of recognizing the word in the images.

## Figures and Tables

**Table 1:** The results of MCAN and BAN are used for the two image features, which are extracted by BUTD and Pythia respectively.

Model	Accuracy(%)
MCAN (Bottom-up image feature)	67.17
MCAN (Pythia image feature)	67.44
BAN-GA (Bottom-up image feature)	66.00
BAN-GA (Pythia image feature)	67.23

**Table 2:** Evaluation of ablation model on VQA v2 validation set. The highest scores are highlighted in bold font.

Ablation Model	Accuracy(%)
BAN-GA	67.23
BAN-GA + BERT	68.83
BAN-GA + BAN-SA	68.81
BAN-GA + Q-SA	68.90
**DMBA-NET(our)**	**69.45**

**Table 3:** Validation scores on VQA2.0 dataset for the number of glimpse of our models. DMBA-NET-L denotes the model has L layer.

Glimpse (Layer)	Accuracy
DMBA-NET-1	69.20
DMBA-NET-2	69.09
DMBA-NET-3	69.30
DMBA-NET-4	**69.45**
DMBA-NET-8	69.36

**Table 4:** Validation scores on VQA2.0 dataset for the BERT’s learning rate.

lr ×	Accuracy
0.001	68.41
0.01	69.30
**0.02**	**69.45**
0.1	69.06

**Table 5:** Comparison with the state-of-the-art approaches on test-dev and test-std of VQA 2.0.

Method	Test-Dev (%)				Test-Std (%)			
	Y/N	Num	Other	Overall	Y/N	Num	Other	Overall
MCB [2]	82.3	37.2	57.4	65.4	-	-	-	-
Bottom-Up [9]	81.82	44.21	56.05	65.32	82.20	43.90	56.26	65.67
Counter [34]	83.14	51.62	58.97	68.09	83.56	51.39	59.11	68.41
MuRel [35]	84.77	49.84	57.85	68.03	-	-	-	68.41
MFB+CoAtt+GloVe+VG [3]	84.1	39.1	58.4	66.9	84.2	38.1	57.8	66.6
Pythia v0.1	-	-	-	68.71	-	-	-	-
MFH [25]	84.27	49.56	59.89	68.76	-	-	-	-
MRA-NET [36]	85.58	48.92	59.46	69.02	85.83	49.22	59.86	69.46
MCAN [11]	86.82	53.26	60.72	70.63	-	-	-	70.9
DFAF [12]	86.73	52.92	61.04	70.59	-	-	-	70.81
CMCN [13]	86.27	53.86	60.57	70.39	-	-	-	70.66
DMBA-NET(our,train+val)	87.55	51.15	60.72	70.69	87.81	50.26	60.79	70.85

## Data Availability

Publicly available datasets were analyzed in this study. Available online: https://visualqa.org/download.html.
